# Ongoing ecological speciation in *Cotesia sesamiae*, a biological control agent of cereal stem borers

**DOI:** 10.1111/eva.12260

**Published:** 2015-07-07

**Authors:** Laure Kaiser, Bruno Pierre Le Ru, Ferial Kaoula, Corentin Paillusson, Claire Capdevielle-Dulac, Julius Ochieng Obonyo, Elisabeth A Herniou, Severine Jancek, Antoine Branca, Paul-André Calatayud, Jean-François Silvain, Stephane Dupas

**Affiliations:** 1 Laboratoire Evolution, Génomes, Comportement et Ecologie, UMR CNRS-Univ. Paris-Sud-IRD, Univ. Paris-SaclayGif-sur-Yvette Cedex, France; 2 INRA, UMR 1392, Institut d'Ecologie et des Sciences de l'Environnement de ParisParis, France; 3 icipe: African Insect Science for Food and HealthNairobi, Kenya; 4 Institut de Recherche sur la Biologie de l'Insecte, CNRS UMR 7261, Université François-Rabelais, UFR Sciences et TechniquesTours, France; 5 Ecologie, Systématique et Evolution, UMR – 8079 UPS-CNRS-AgroParisTech, Univ. Paris-SudOrsay Cedex, France

**Keywords:** adaptation, Africa, cryptic species, ecological niche, evolution, geographic distribution, host range, Hymenoptera, parasitic wasp, phylogeny, reproductive isolation, virulence

## Abstract

To develop efficient and safe biological control, we need to reliably identify natural enemy species, determine their host range, and understand the mechanisms that drive host range evolution. We investigated these points in *Cotesia sesamiae*, an African parasitic wasp of cereal stem borers. Phylogenetic analyses of 74 individual wasps, based on six mitochondrial and nuclear genes, revealed three lineages. We then investigated the ecological status (host plant and host insect ranges in the field, and host insect suitability tests) and the biological status (cross-mating tests) of the three lineages. We found that one highly supported lineage showed all the hallmarks of a cryptic species. It is associated with one host insect, *Sesamia nonagrioides,* and is reproductively isolated from the other two lineages by pre- and postmating barriers. The other two lineages had a more variable phylogenetic support, depending on the set of genes; they exhibited an overlapping and diversified range of host species and are not reproductively isolated from one another. We discuss the ecological conditions and mechanisms that likely generated this ongoing speciation and the relevance of this new specialist taxon in the genus *Cotesia* for biological control.

## Introduction

Using the natural enemies of crop pests is a common method of biological control (Bale et al. 2008). This sustainable agricultural practice is increasing worldwide in response to public concern about the use of chemical products and genetically modified crops. The United Nations considers biological control to be an effective ecosystem service (Millennium Ecosystem Asessment 2005). However, the efficient and safe use of biological control agents requires their reliable identification and the accurate determination of their host range (Rosen 1986; Brodeur 2012). Direct and indirect effects on nontarget host populations have been documented (e.g., reviews by Bigler et al. 2006; De Clercq et al. 2011), so many countries now regulate the import and use of biological control agents (Hunt et al. 2008; EPPO 2010).

Natural enemies specialized on the targeted pest are thus relevant in biological control. Many species may appear generalist but careful ecological studies may reveal that they are an assemblage of populations with more restricted host range. Misidentification of populations has caused cases of failures of biological programs, when the introduced population was unable to prey on or parasitize the targeted pest (e.g., Mohyuddin et al. 1981; Gitau et al. 2007). The use of specialized natural enemies is a prerequisite to avoid effects on nontarget hosts, but the stability of host range is not guaranteed. In insect parasitoids or predators, which include major biological control agents and limit insect populations in the wild (Hawkins 1994), host preference may be learned and thus may be plastic (Kester and Barbosa 1991; Davis and Stamps 2004; Kaiser et al. 2009). Or it may be genetically determined and stability of host preference will then depend on the amount of gene flow between host specialized populations, sometimes referred to as host races. Stable host preference is expected when there is no more gene flow in the case of host races that have differentiated into separate species (ecological speciation). Alternatively, depending on ecological conditions, conserved gene flow among specialist populations could maintain their ability to shift to nonpreferred hosts, if preferred hosts become scarce (Mochiah et al. 2002; Baer et al. 2004). To assess the evolutionary stability of host range, we need to combine ecological, phylogenetic, and experimental approaches to identify the mechanisms that generate and maintain specialization (Hufbauer and Roderick 2005).

There has been extensive research into ecological specialization and speciation in phytophagous insects (reviewed by Dres and Mallet 2002; Futuyma 2008), but less is known in parasitoids which are difficult to sample due to low population densities in the wild and difficulties of rearing in the laboratory. Several species identified morphologically were considered initially as generalists and have now been split into closely related, more specialized species, based on the inclusion of molecular and ecological data (e.g., Smith et al. 2006; Heraty et al. 2007; Phillips et al. 2008; Hambäck et al. 2013). These are defined as cryptic species (Bickford et al. 2006). However, little is known on the mechanisms that generated and maintained specialization: Which traits underwent divergent selection? Which mechanisms insured reproductive isolation? Among candidate traits, the evolution of virulence mediated by symbiotic poly-DNA viruses (PDVs) has been well documented (Pennacchio and Strand 2006; Branca et al. 2012; Herniou et al. 2013). Several PDV virulence genes have been identified (Bitra et al. 2011; Bézier et al. 2013) and adaptive selection on some of them drove the specialization of *Cotesia* parasitoid wasps (Herniou et al. 2013).

*Cotesia sesamiae* (Cameron) (Hymenoptera: Braconidae) belongs to the *C. flavipes* monophyletic complex that is made up of four allopatric sister species (Kimani-Njogu and Overholt 1997; Muirhead et al. 2012). They are gregarious endoparasitoids of lepidopteran stem borers of Crambidae, Pyralidae, and Noctuidae families. Members of the complex are economically important worldwide as biocontrol agents of cereal and sugarcane stem borer pests (Polaszek and Walker 1991). *C. sesamiae* is the African species of the complex, with a sub-Saharan distribution. This generalist species (Mailafiya et al. 2010) is the main larval parasitoid of a major African maize pest, the noctuid moth *Busseola fusca* (Fuller) (Kfir et al. 2002). It was introduced successfully to Madagascar and Mauritius to control cereal stem borers (the noctuid *Sesamia calamistis* Hampson, and the crambid *Chilo partellus* (Swinhoe), Overholt 2000). Phylogenetic analyses of the *C. flavipes* species complex, based on two mitochondrial and three nuclear genes, did not support host specialization of the different lineages (Muirhead et al. 2012), possibly because samples came from cultivated plants on which only a few host species are found. To overcome this limitation, *C. sesamiae* has been collected from various hosts on wild and cultivated plant species, based on extensive, multiyear sampling in several countries across sub-Saharan Africa. Genetic analyses revealed that populations specialized on different lepidopteran host genus harbored distinct allelic variants of the PDV virulence gene *CrV1* (involved in the inactivation of host hemocytes in *Cotesia rubecula*, Asgari and Schmidt 2002) and that the evolution of this virulence gene explained partly the host range (Gitau et al. 2007; Dupas et al. 2008; Branca et al. 2011). Working on a subsample of *C. sesamiae* obtained from twenty different associations of host insects and plants, Jancek et al. (2013) reported genetic differentiation of, and positive selection on two additional viral genes (histone 4 and EP2 involved in inhibiting the caterpillar's immune responses, Gad and Kim 2008; Kwon and Kim 2008), with partial correspondence with *CrV1* lineages.

This study aimed to investigate whether the reported ecological specialization of *C. sesamiae* corresponds to distinct lineages and whether they represent cryptic species. We use some of Branca et al. (2011) *C. sesamiae* samples along with newly added insects from other combinations of hosts, plants, and localities. We genotype mitochondrial and nuclear viral and nonviral genes to establish whether wasps found on the same host species are phylogenetically related. We then investigate the geographic distribution and ecological specialization of the identified lineages in the field and conduct a reciprocal transfer experiment with samples of each of these lineages to test whether host use had an adaptive component. We then test reproductive isolation by cross-mating experiments between laboratory strains from each lineage, and with another species of the *flavipes* complex (*C. flavipes*). Finally, we discuss the species status of one of the identified lineages that specializes on the moth *Sesamia nonagrioides* (Lefèbvre), a major maize pest in Mediterranean countries.

## Materials and methods

### Insect sampling

Stem borer larvae were collected from wild plants at 37 sites in six countries of eastern sub-Saharan Africa (Table S1), using the sampling scheme for natural habitats described by Le Ru et al. (2006). At each site, wild plants were examined in the following habitats when present (i) in and around crops, (ii) in open patches along forest roads, (iii) on banks of streams or rivers, and (iv) in swamps. We used a selective sampling procedure rather than a random one to increase the chance of finding stem borers that are at lower densities on wild host plants than on the adjacent cultivated cereals (Ong'Amo et al. 2006). In all habitats, plant species belonging to the Poaceae, Cyperaceae, and Typhaceae were carefully inspected for stem borer infestations. These are the main families on which Crambidae and Noctuidae stem borer larvae (the hosts for species of the *flavipes* complex) prefer to feed in the study region. Symptoms of infestation included scarified leaves, dry leaves and shoots (dead hearts), frass, or holes bored. Infested plants were cut and dissected in the field.

Stem borer larvae were identified at least to family or to species using a larval picture library from the IRD (Institut de Recherche pour le Développement) and information about host plant assemblages, as most stem borers are host-plant-specific (Le Ru et al. 2006). Adult moths were identified by dissection of the genitalia. Larvae collected from the field were reared on an artificial diet (Onyango and Ochieng'-Odero 1994) until pupation or emergence of parasitoid larvae. After emergence, adult parasitoids were stored in absolute ethanol. Morphological identification of parasitoids was based on genitalia shape (Kimani-Njogu and Overholt 1997).

### Phylogenetic analysis

A total of 74 *C. sesamiae* individuals were sequenced for six genes: three mitochondrial genes (16S rRNA, COI, and NADH1) and three nuclear genes including two polydnaviruses (early expressed protein or EP2, and histone 4) and one nonviral gene (long-wavelength rhodopsin or LWRh). Primers, references, sequence length, PCR conditions, and sequence accession numbers are detailed in Table S2. Five *C. flavipes* and three *C. chilonis* samples were also sequenced for the same genes. *C. congregata* (Say) was added as an outgroup using sequences for the six genes from GenBank. The accession numbers are HQ552539 (COI), DQ538528 (16S), AF069198 (NADH1), HF586473 (EP2), HF586475 (histone 4), AJ535980, and DQ538700 (exons 1 and 2 of LWRh).

Phylogenetic reconstructions were performed on the whole dataset with partitions and also independently for each group of genes. The software PartitionFinder (Lanfear et al. 2012) was used to determine the best subset of partitions. The tested partitions were based on the different genes and on codon positions for the coding genes of the dataset. The best-fit model of substitution for each partition was determined using the Bayesian information criterion. The phylogenetic relationships were estimated with Bayesian inference using the program MrBayes v3.2.1 (Ronquist and Huelsenbeck 2003). The run consisted of two independent analyses with the following settings: four Markov chains of twenty million generations, random starting trees, default priors, and trees sampled every 100 generations (branch lengths were also saved). A burn-in period of four million generations was used. Node support was estimated by clade posterior probability (CPP). The PSRF (potential scale reduction factor) and ESS (effective sample size) values were checked to make sure convergence was reached. All PRSF values were equal to 1, and ESS values were all above 200, which indicates the convergence of the runs.

### Ecological specialization

The family and species status of the host insects and the host plants were reported on the phylogenetic tree. For a better understanding of the observed differences in host range between *C. sesamiae* lineages, we characterized the diversity of host insects and plants in the sampled sites, by analyzing the relative abundance of stem borer species collected on the different plant tribes for each pool of sites where specimens of a given lineage of *C. sesamiae* had been collected (Fig. 3). This formed three pools of sites, one per lineage (over the 37 sampled sites, only one hosted *C. sesamiae* samples from two lineages).

### Reciprocal transfer experiments

To determine whether host ranges corresponded to specific adaptation, we measured the reproductive success of three *C. sesamiae* laboratory strains, one within each of the three observed lineages in the phylogenetic analysis (see Results), when parasitizing their own native noctuid stem borer species and when transferred to the native hosts of the other two strains. These host species were *B. fusca, S. calamistis,* and *S. nonagrioides*. The *C. sesamiae* laboratory strains originated from the following locations in Kenya: Kitale (34.818E, 1.1956N) for strain Cs Kitale (sample CsK, lineage 1), Makindu (37.825E, −2.278S) and Mbita Luanda (34.2973E, −0.4833S) for strain Cs Typha (samples Mbl and Mkd, lineage 2), and Mombasa (39.667E, −4.05S) for strain Cs Mombasa (sample MhK, lineage 3). Cs Kitale was reared on *B. fusca*, Cs Mombasa on *S. calamistis*, and Cs Typha on *S. nonagrioides*. The host caterpillars were fed an artificial diet at 26°C (following Overholt et al. 1994).

Reciprocal transfer experiments were performed as follows. Three weeks after eggs hatched, host larvae from the three species were taken from rearing vials and placed on fresh pieces of maize stem for 24 h, to ensure acceptance by the parasitoid (Overholt et al. 1994). They were parasitized individually by a single *C. sesamiae* female and placed in a Petri dish with a piece of maize stem and a piece of wet paper, under the same prior rearing conditions, until observation of either (i) the formation of parasitoid cocoons, (ii) death of the host larvae, without cocoon formation, or (iii) formation of host pupa. The proportion of host larvae allowing parasitoid cocoon formation was used as an estimator of the parasitoid's reproductive success. Each host species was exposed to all three *C. sesamiae* strains. A chi-square test was conducted to compare proportions of reproductive success (using XLStat software from Addinsoft, Paris, France, with application of a Yates correction for continuity when df = 1).

### Reproductive isolation

Reproductive isolation tests were performed by crossing Cs Typha strain with Cs Kitale, Cs Mombasa, and *C. flavipes*. These strains are naturally infected with different *Wolbachia* strains that cause reproductive isolation (Branca et al. 2011), so they were treated to eliminate the bacteria before experiments commenced (see Appendix S1). Crosses between Cs Kitale and Cs Mombasa were performed in previous studies showing that these strains can interbreed in one direction of cross (Mochiah et al. 2002; Gounou et al. 2008; Branca et al. 2009, 2011). They are not interfertile with the species *C. flavipes* (Kimani-Njogu and Overholt 1997). Precopulatory isolation was determined from observations of mating behavior, and postcopulatory isolation, from progeny data.

For mating observations, cocoons were isolated when turning gray, as the blackish color of forming adult was visible through the cocoon silk. They were placed in 2.5-mL plastic vials with a droplet of 5% saccharose water solution, at 60% RH and at 21 or 25–26°C, to synchronize adult emergence between the two strains to be crossed. Mating was observed at 0–2 days following emergence. Each couple was enclosed in a small plastic vial (1 cm diameter × 2 cm height) to record the occurrence, latency up to 35 min, and duration of copulation. Mated females were then placed in 2.5-mL vials under rearing conditions (25°C, 60% RH), for 24 h until parasitism began. Control and hybrid matings were observed on the same day.

We allowed each mated female to oviposit following the protocol described in the above section ‘Reciprocal transfer experiments’. Parental females were tested on *S. nonagrioides* for Cs Typha, *S. calamistis* for Cs Kitale and Cs Mombasa, and *C. partellus* for *C. flavipes*, and hybrid females were tested on maternal and paternal hosts. Clusters of the resulting cocoons were transferred in clean vials and kept under rearing conditions until adult emergence. Parasitoid development was quantified by the percentage of parasitized host larvae that produced a cocoon cluster (% cocoon clusters). The progeny traits measured were progeny size (number of males, females, and nonhatched cocoons in each cluster), nymphal mortality, and sex ratio (number of females divided by number of adults). From the resulting progeny, first hybrid generation females (F1) were crossed with F1 males (equivalent to males of the maternal strain, as they are haploid) to estimate the probability of getting a second hybrid generation.

Proportions were compared using a chi-square test or a Fisher exact test when the average expected frequency was below 6 (Zar 1999). A Kruskal–Wallis test was used to compare continuous data because they were not distributed normally (based on a Shapiro–Wilk test). In the case of significant variability between groups, values were compared by multiple pairwise comparisons using a Marascuilo or Dunn test to compare proportions and quantitative traits, respectively. Based on the data for mating occurrence (mating probability), parasitic development (probability of cocoon formation), and progeny traits (probability of nymphal mortality and of female progeny), we calculated the expected net reproductive rate (expected number of daughters per mother) as follows:


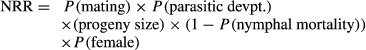


## Results

### Phylogenetic analyses and ecological and geographical distributions

**Figure 1:**
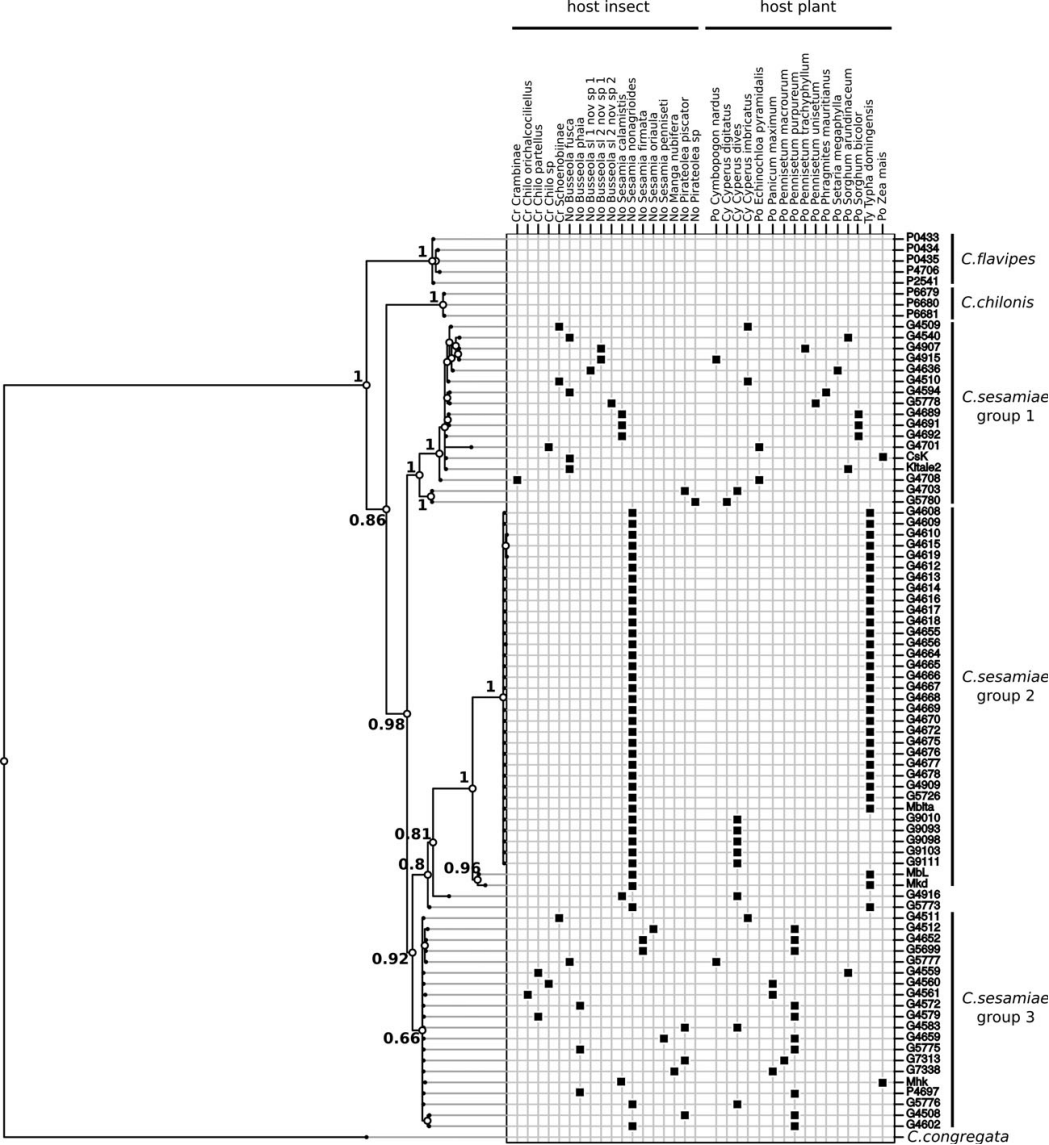
Phylogeny of *Cotesia sesamiae* individuals and relatives based on concatenated mtDNA of 3 genes (CO1, 16S, and NADH) and nDNA of a nonviral (LWRH) and two viral genes (EP2 and histone) in relation to host insect and host plant species matrix. See Materials and methods for substitution model selection with PartitionFinder and phylogenetic tree inference in Mr Bayes. Posterior probabilities are given at nodes. All samples have a reference code corresponding to the data bank of the Laboratoire Evolution, Génomes, Comportement et Ecologie. Insect family: Cr, Crambidae; No, Noctuidae. Plant family: Po, Poaceae; Ty, Typhaceae; Cy, Cyperaceae.

Phylogenetic reconstructions obtained from the all-gene dataset, or for mtDNA+LWRH or PVD genes independently, strongly supported the monophyly of the three sister species in the *C. flavipes* complex, with posterior probability ranging from 0.98 to 1 and evidenced the relationship *C. flavipes* (*C. chilonis*, *C. sesamiae*) (Fig. 1: mtDNA+LWRH+PDV; Fig. S1A: mtDNA+LWRH; Fig. S1B: PDV). The mtDNA genes provided a lower support to the *C. sesamiae* lineage, and the LWRH gene failed to resolve relationships within the *flavipes* complex (Table 1).

**Table 1:** Phylogenetic support (Bayesian posterior probability) of the *Cotesia sesamiae* lineages for each gene partition

Genes	Length of concatenated sequence (bp)	Lineage *C. sesamiae*	Lineage 1	Lineage 2	Lineage 2 + G5773	Lineage 3
mtDNA+ LWRH+ PDV	2756	0.98	1	1	0.8	0.66
mtDNA+ LWRH	1877	0.87	1	1	0.92	0.80
PDV	879	0.75	–	0.95	–	0.72*

mtDNA, 16S rRNA, COI and NADH1; PDV, poly-DNA virus nuclear genes EP2 and histone 4; LWRh, long-wavelength rhodopsin (nonviral nuclear DNA);–, no such lineage/group.

* In this phylogeny, samples G4708, 4703, 5780 are in lineage 3 instead of being in lineage 1 in the other phylogenies.

Within *C. sesamiae,* analysis revealed the existence of three lineages (Figs 1 and S1). Lineage 1 was defined with strong support (posterior probability of 1), except for the analysis of PDV genes (Table 1, Figs 1 and S1). It included 17 samples found on a variety of host insects and host plants (Fig. 1, Table 2). A second lineage with a 0.92 support value combined two individuals (G4916 and G5773), a sublineage composed of 35 samples all collected from *S. nonagrioides* on two plants *Typha domingensis* Pers. (Typhaceae) and *Cyperus dives* Delile (Cyperaceae) (Fig. 1) and another sublineage that comprised 19 samples from several host insects and host plants (Fig. 1, Table 2). These two ‘sublineages’ were numbered lineages 2 and 3, respectively. Lineage number 2 (comprised of individuals found on *S. nonagrioides*) was supported by a posterior probability of 1 (Table 1). Lineage number 3 had more variable support, ranging from 0.66 to 0.80 (Table 1). The two samples (G5773 and G4916) that were found outside of the three main lineages had phylogenetic relationship with the lineages that depended on the partition (Fig. S1). Three other samples that fell in lineage 1, according to the all-gene and neutral gene datasets, were assigned to lineage 3 based on the PDV genes.

**Table 2:** Width of insect and plant host ranges for *C. sesamiae* lineages identified on Fig. 1

Lineage	*N*	No. of insect hosts	No. of plant hosts	No. of insect–plant genus assoc.
1	17	8 spp./4 genera	12 spp./9 genera	10
2	35	*S. nonagrioides*	2 spp./2 genera	2
3	20	11 spp./6 genera	8 spp./7 genera	12
Total range		15 spp./7 genera	15 spp./7 genera	19
Shared 1-3		4 spp./4 genera	5 spp./5 genera	4
Shared 2-1		0	1	0
Shared 2-3		1	1	1

There was overlap in about half of the range of host insects, and host plant species, of lineages 1 and 3 (Table 2). Parasitoids were found on the same four genera of host insects—*Chilo* (Crambidae), *Busseola*, *Pirateolea*, and *Sesamia* (Noctuidae)—except for one sample of lineage 3 found on a *Manga* sp. (Noctuidae). Samples of lineages 1 and 3 came from a variety of wild plants belonging to the main three families that host the lepidopteran stem borers parasitized by *C. flavipes* complex: Cyperaceae, Poaceae, and Typhaceae. No samples from these two lineages were found on *S. nonagrioides* or *T. domingensis*, while one sample positioned close to lineage 2 (G5773, Fig. 1, Table 1) was collected on this association.

**Figure 2:**
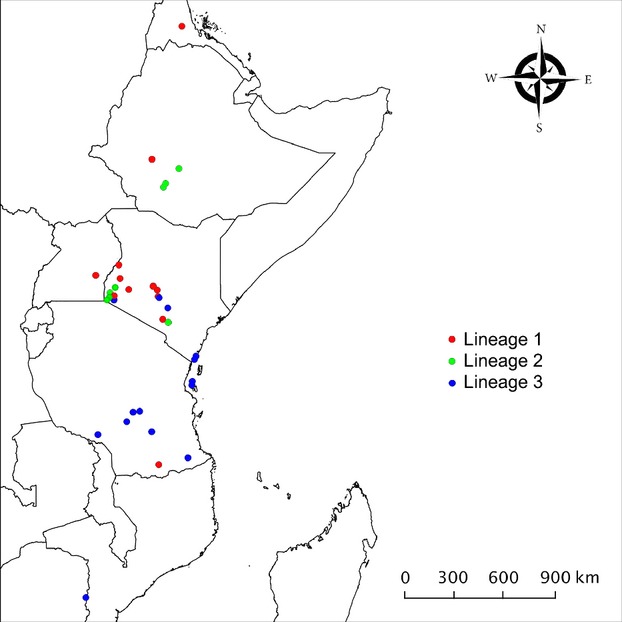
Geographical distribution of *C. sesamiae* samples in sub-Saharan East Africa. Lineages 1, 2, and 3 are lineages defined by the phylogenetic analysis (Fig. 1).

Geographically, all samples were collected in the eastern part of *C. sesamiae*'s range (Fig. 2), which covers sub-Saharan Africa from Cameroon, east toward the Indian Ocean, and from Eritrea, south toward the Republic of South Africa (Polaszek and Walker 1991). Although all three lineages were found in Kenya, sometimes in close proximity, distributions of lineage 1 and 3 were different. The samples in lineage 1 were found in 12 sites distributed in west Kenya, Uganda, Ethiopia, Erytrea, and Tanzania. With the exception of the Tanzanian site, these sites were located mostly north and west of samples in lineage 3, which were found in 16 sites distributed in south Kenya, Tanzania, Zanzibar, and Mozambic. One of this site (Ruiru, central Kenya) hosted samples from lineage 1 and 3. Samples in lineage 2 were found in fewer locations (8) distributed in south and west Kenya, close to lineage 1 and 3 sites, and in Ethiopia, close to lineage 1 site.

### Ecological niche of *C. sesamiae* lineages

**Figure 3:**
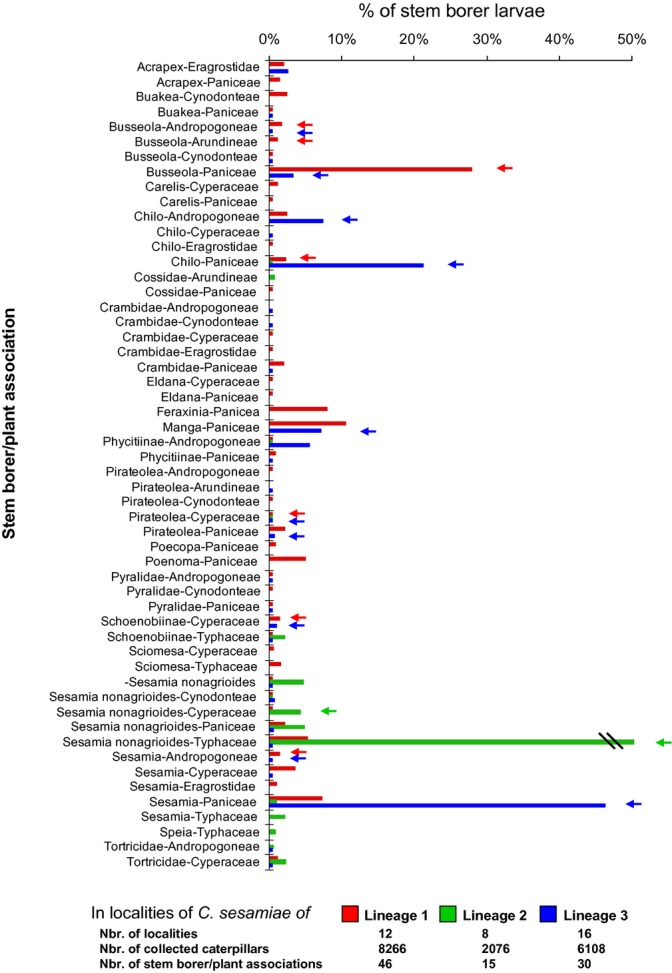
Relative abundance of stem borer–plant associations present in the sites where *C. sesamiae* samples were found. Sites hosting *C. sesamiae* from the same lineage were pooled for the analysis. Arrows indicate on which association *C. sesamiae* samples were found, and colors correspond to the lineage.

To better understand the observed differences of host range between *C. sesamiae* lineages, we characterized the diversity and relative abundance of host insects and plants in the sampled sites. Although we considered stem borer genus level for this analysis, we kept *S. nonagrioides* as separate species because of its particular association with lineage 2. Lineages 1 and 3 sites shared many associations of stem borer genus and their host plant tribes, but did not have the same dominant association (Fig. 3). *Busseola* stem borers on Paniceae were dominant in lineage 1 sites, whereas *Sesamia* (not *nonagrioides*) and *Chilo* stem borers on Paniceae were dominant in lineage 3 sites. The sites hosting samples of lineage 2 were characterized by fewer associations. In these sites, the association of *S. nonagrioides* on *Typha domingensis* was the most abundant, whereas it was weakly represented or rare in the other sites. Regarding the presence of *C. sesamiae*, the samples from lineages 1 and 3 were found on various dominant and rare associations, whereas samples from lineage 2 were found only on two riparian associations, one largely dominant: *S. nonagrioides* on Typhaceae, and the second less common: *S. nonagrioides* on Cyperaceae, although eight stem borer genera were present. *S. nonagrioides* was the most abundant species on both plant families (Fig. 3).

The genetic differentiation of lineage 2 associated with ecological specialization may correspond to divergent selection for parasitic success on a given host species. The reciprocal transfer experiments were therefore used to assess adaptation to host species in the three observed lineages.

### Reciprocal transfer experiments

The measurements of reproductive success (Table 3) showed that Cs Kitale (lineage 1) was the only strain that oviposited on *B. fusca* larvae, and it had a similar probability of producing progeny in *B. fusca* or *S. calamistis* (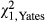 = 0.03; *P* > 0.5). Cs Typha (lineage 2) was almost the only strain able to develop in *S. nonagrioides*. It had a higher probability of producing progeny in *S. nonagrioides* than in *S. calamistis* (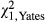 = 7.34; *P* < 0.01). Cs Kitale (lineage 1) and Cs Mombasa (lineage 3) oviposited readily on *S. nonagrioides* larvae, but most parasitized caterpillars survived the parasitism and formed pupae. Cs Coast was able to develop only in *S. calamistis*, which was an equally suitable host species for the three parasitoid strains (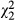 = 4.24; *P* > 0.5).

**Table 3:** Reciprocal transfer experiments: reproductive success on different host species

	Host species			
*C. sesamiae* strains	*B. fusca*	*S. nonagrioides*	*S. calamistis*	*N*
Cs Kitale (lineage 1)	65	5	68	60/37/114
Cs Typha (lineage 2)	0 (no sting)	66	45	53/176/60
Cs Mombasa (lineage 3)	0 (no sting)	0	77	40/30/115

*N*, respective numbers of host larvae parasitized; no sting, wasps did not attempt to parasitize host larvae.

Percentages of host larvae that exhibited successful parasitic cocoon formation.

### Reproductive isolation

#### Crosses between the strains Cs Typha and Cs Mombasa

The success of crosses between strains depended on the direction of the cross: few Mombasa females mated with Typha males (M×T), but not significantly less than with Mombasa males, which reflected a reduced probability of mating among these females, in our laboratory conditions. The probability of parasitic development was not different from what was observed in the parental strains, but female progeny were rare, so the resulting net reproductive rate of this first hybrid generation was close to 0 and consequently not tested at the second generation (Table 4A).

**Table 4:** Test of reproductive isolation between strains from the three *C. sesamiae* lineages and *C. flavipes* using cross-mating experiments

	Mating occurrence		Mating traits			Parasitic devpt.		Progeny traits				
(♀ × ♂)	*n*_obs_	% couples mating	*n*_mated_	Latency	Duration	*n*_hosts_	% cocoon clusters	*n*_cluster_	Size (no. of cocoons)	Nymphal mortal. (%)	Sex ratio (%♀)	Resulting net reprod. rate
A. Crosses between Cs Typha strain (lineage 2) and Cs Mombasa strain (lineage 3)												
T × T	30	77 a	23	472 (113) ab	21 (1) a	20	80	16	52 (9)	9 (3)	47 (9) a	10 (3) a
M × M	60	33 b	20	536 (177) ab	23 (4) a	20	90	18	50 (5)	11 (6)	74 (7) a	9.5 (2) a
M × T	56	16 b	9	74 (28) b	23 (3) ab	9	89	8	58 (7)	7 (4)	0.005 (0) b	0.05 (0.04) b
T × M	38	66 a	25	660 111) a	41 (5) b	14	71	10	48 (11)	14 (6)	37 (12) a	5 (3) a
analysis		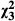 **= 36.62** ***P*** **<10**^**−4**^		***K***_**3**_ **= 9.82** ***P*** **=0.020**	***K***_**3**_ **= 13.38** ***P*** **=0.004**		Fisher test *P =* 1, NS		*K*_3_ = 1.06 NS	*K*_3_ = 3.36 NS	***K***_**3**_**=21.21** ***P*** **<10**^**−4**^	***K***_**3**_**=16.88** ***P*** **=10**^**−3**^
TM × T	53	51	27	234 (89)	16 (1)	10/17	0/23.5	4	2.5 (0.3)	54 (16)	100	0/0.15
TM × M	67	72	48	474 (77)	28 (2)	11/24	0/68	16	11 (2)	33 (9)	99	0/4.5
B. Crosses between Cs Typha strain and Cs Kitale strain (lineage 1)												
T × T	34	59 a	20	536 (154)	22 (1) a	17	71	12	62 (7) ab	16 (7)	43 (9) a	9 (2) a
K × K	29	69 a	20	383 (104)	18 (1) a	19	58	11	53 (7) a	6 (3)	67 (8) a	14 (3) a
K × T	30	0 b	0	///	///	///	///	///	///	///	///	0
T × K	31	84 a	26	631 (144)	57 (5) b	21	48	10	84 (10) b	8 (6)	3.3 (2) b	1 (0.6) b
analysis		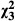 **= 49.15** ***P*** **<10**^**−4**^		*K*_2_ = 0.75 NS	***K***_**2**_**= 36.70** ***P*** **<10**^**−4**^		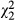 = 2.03 NS		***K***_**2**_ **= 7.05** ***P*** **=0.03**	*K*_2_ = 3.28 NS	***K***_**2**_**=18.81** ***P*** **<10**^**−4**^	***K***_**2**_**=15.63** ***P*** **<10**^**−3**^
TK × T	48	75	36	284 (60)	17 (1)	13/15	0/7	1	4	100	///	0
TK × K	35	94	33	284 (68)	56 (6)	15/9	7/11	1/1	18/1	17/0	100/100	1/0.1
C. Crosses between Cs Typha strain and *C. flavipes*												
T × T	124	60 a	75	427 (36) c	24 (1) b	48	63	30	33 (2) ab	11 (1) a	54 (5) b	6 (1) a
Cf × Cf	112	62 a	69	201 (25) a	18 (1) a	55	42	23	40 (4) a	7 (2) a	80 (6) a	8 (1) a
T × Cf	50	72 a	36	384 (83) ab	43 (5) c	28	43	12	26 (4) b	12 (2) a	0 (0) c	0 (0) b
Cf × T	163	7 b	12	458 (93) bc	14 (2) a	12	42	5	37 (5) ab	7 (2) a	0 (0) c	0 (0) b
Analysis		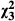 **= 133** ***P*** **<10**^**−4**^		***K***_**3**_ **= 35.72** ***P*** **<10**^**−4**^	***K***_**2**_ **= 105.3** ***P*** **<10**^**−4**^		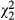 = 5.32 NS		*K*_3_ = 8.05 *P* = 0.045	*K*_3_ = 8.08 *P* = 0.045	***K***_**3**_**=43.10** ***P*** **<10**^**−4**^	***K***_**3**_**=33.81** ***P*** **<10**^**−4**^

T, Cs Typha strain; M, Cs Mombasa strain; K, Cs Kitale strain; TK, hybrid daughters from T mother and K father; TM, daughters from T mother and M father; *n*, sample size.

See Materials and methods for significance of traits and statistical analyses.

Mean values and standard errors (in brackets) are given for each trait. Gray highlight is values indicating reproductive barriers. Parental females were tested on their developmental host, *S. nonagrioides* for Cs Typha, and *S. calamistis* for Cs Kitale and Mombasa. Hybrid females were tested on *S. nonagrioides* and *S. calamistis* (left/right, respectively). Letters indicate significant difference at *P* < 0.05. Statistical results are in bold when significant.

In the reciprocal cross (T×M), the various traits were not significantly different from those in the control crosses, except that mating duration doubled, with the male having observed difficulty disengaging from the female. Hybrid F1 daughters (labeled TM) were backcrossed with males of both parental lines, and their progeny did not develop in *S. nonagrioides*. In *S. calamistis,* parasitic development occurred but progeny traits depended on the male parental strain. Hybrid females crossed with Typha males produced very few cocoons that contained only females. The reciprocal backcross gave more abundant but also all-female progeny (except for one male). After two generations, there was a low probability of obtaining a hybrid lineage between Typha and Mombasa strains.

#### Crosses between the strains Cs Typha and Cs Kitale

Kitale females did not mate with Typha males; the males performed courtship behavior that elicited no response from females, which were thus not further tested for the production of progeny (Table 4B).

In the reciprocal cross, the probability of mating was not different than that in the parental strains, but copulation lasted two to three times longer, due to males' difficulty in disengaging from females. The probability of parasitic development was not different than in the parental strains, but there were very few female offspring, so the expected net reproductive rate was about 10-fold lower than in parental strains.

F1 females were backcrossed with males of both parental lines. The probability of parasitic development was low (from 0 to 11%) in both *S. nonagrioides* and *S. calamistis*. A total of 52 females produced only three small cocoon masses, resulting in no male progeny and a net reproductive rate between 0 and 1, depending on the backcross and the host species. After two generations, the probability of obtaining a hybrid lineage was close to zero.

#### Crosses between Cs Typha strain and *C. flavipes*

Both directions of hybrid crosses produced no female progeny, indicating systematic mortality of fertilized eggs. Mating problems were also observed: *C. flavipes* females rarely mated with Typha males, as male courtship behavior elicited no response from females. In the reciprocal cross, mating occurrences were not different than in the parental strains, but mating duration was about double, again with the male having observed difficulty disengaging from the female. Mating latency was significantly shorter when the male was *C. flavipes* in the control and between species crosses (Table 4C).

## Discussion

Our results revealed that generalist and specialist lineages of *C. sesamiae* coexist. The support of two lineages (numbers 1 and 3) depended on the type of genes used in phylogenetic reconstruction. Both lineages were somewhat generalists and shared part of their host ranges, and strains of each lineage were known to be able to interbreed. They may correspond well to the two lineages of *C. sesamiae* revealed by the analyses of phylogenetic relationships within the *C. flavipes* complex by Muirhead et al. (2012), based on two *mt* genes that were also included in our analysis. Muirhead et al.'s two lineages of *C. sesamiae* showed similar geographical differentiation to ours, with one having a more north-western distribution (west Kenya) than the other (east Kenya and countries of southern Africa). The genetic separation between lineages 1 and 3 could then be explained by a known past geographical barrier to gene flow in that part of Africa — the Oriental Rift Valley — that is known to have influenced genetic differentiation in many taxa (e.g., Sezonlin et al. 2006).

Presently, lineages 1 and 3 can currently be found in the same geographic area. This may be because changes in land use can alter the spatial availability of host insects and plants, which in turn can cause range expansions or restrictions. Limited gene flow between lineages 1 and 3 is maintained by a *Wolbachia*-induced reproductive barrier, because samples of the two lineages are infected by distinct *Wolbachia* strains, which causes cytoplasmic incompatibilities (Mochiah et al. 2002; Gounou et al. 2008; Branca et al. 2009, 2011). Local adaptation could also partly explain the genetic differentiation between lineages 1 and 3. *Busseola* was dominant in sites where *C. sesamiae* from lineage 1 were found, and only samples of this lineage were virulent against *B. fusca. Sesamia* (not *nonagrioides*) was dominant in lineage 3 sites. Lineages 1 and 3 may then correspond, at least partially, to the Inland (west Kenya) and Coast (east Kenya) host races identified by Dupas et al. (2008). Inland host race is virulent against *B. fusca* and the Coast host race is not, which is associated with a differentiation of the virulence gene CrV1 (Gitau et al. 2007, 2010; Branca et al. 2011). Together, our results support the conclusion that lineages 1 and 3 are genetically differentiated because of a geographic barrier and that they are locally adapted to the most abundant host species. However, they are not cryptic species because they can cross.

Lineage 2 received strong support in the phylogenies reconstructed from the different gene datasets. It was also differentiated for the CrV1 gene—we checked that many samples had the ‘Snona’ allele known to characterize *C. sesamiae* collected on *S. nonagrioides* (Branca et al. 2011). Using microsatellite markers, Branca et al. also found that this host race was genetically distant from other *C. sesamiae* clusters. Our study showed further that this host race was found mainly on the insect–plant association, *S. nonagrioides–T. domingensis*, by far the most abundant among all combinations present at the sampling locations. Reciprocal transfer experiments confirmed its unshared virulence on *S. nonagrioides,* which can be interpreted as the result of divergent selection, and confirmed local adaptation to this abundant resource. Results from crossing experiments indicated pre- and postmating incompatibilities between a laboratory strain in lineage 2 and laboratory strains in lineages 1 and 3. These reproductive barriers were associated with a loss of fertility and of virulence in the rare hybrid females, precluding a hybrid lineage and showing that natural selection had occurred in response to maladaptive hybridization. So the differentiation of lineage 2 may well correspond to a case of ecological speciation (Faria et al. 2014).

We were also interested in when and how a parasitoid population would evolve as a specialist entity within a generalist species. The spatial and temporal availability of plant–stem borer associations provides clues for understanding when specialization may confer a selective advantage. Species of Typhaceae are perennial plants that inhabit humid areas. In sub-Saharan Africa, they often form large uniform groups and harbor few stem borer species. Cyperaceae plants often interpenetrate *Typha* settlements, which may explain the presence of both *S. nonagrioides* and *C. sesamiae* on this plant tribe. Availability of the *S. nonagrioides–T. domingiensis* resource may thus confer a selective advantage to parasitoids that are able to counter the host resistance, which may then enable them to evolve as a specialist entity. In *C. sesamiae,* reproductive isolation from other ecological populations is indeed possible without geographical barrier because it is favored by sib-mating in the host tunnel (Branca et al. 2009), short adult life expectation (Potting et al. 1997; Muirhead et al. 2010), low population densities, weak dispersal abilities (Omwega et al. 2006), and Wolbachia infection (Branca et al. 2011).

Wild Poaceae species that host stem borer species parasitized by generalist lineages of *C. sesamiae* grow in a diversified pattern with other Poaceae species; their availability is seasonal and they harbor a more diverse community of stem borer species than do Typhaceae (Le Ru et al. 2006). The seasonal character of these host species would confer a selective advantage to generalist parasites and counterselect strict host specialization. In *C. sesamiae,* the Inland host race has evolved virulence against *B. fusca* but can develop on *S. calamistis* with equal reproductive success. This absence of strict ecological specialization confers a selective advantage because the parasitoids are adapted to *B. fusca*, which can be locally and temporally dominant (Ong'Amo et al. 2006; Dupas et al. 2008; Calatayud et al. 2014), but can shift to other hosts when or where *B. fusca* becomes rare.

Results from phylogenetic analysis, ecological data, and observations of reproductive isolation are thus consistent and indicate an ongoing process of ecological speciation in the lineage of *C. sesamiae* specialized on *S. nonagrioides* on two associated riparian plants*, T. domingensis* and *Cyperus dives*. So far, the *flavipes* complex includes four allopatric species. One of them, *C. nonagriae* (Olliff), was recently removed from synonymy with *C. flavipes* (Muirhead et al. 2008). It is the Australian member of the complex, and the first recorded host was a *Nonagria* noctuid. The lineage specialized on *S. nonagrioides* may become a fifth species of the complex, and the first documented case of ecological speciation in this complex. Morphological analysis must still be carried out. This lineage 2 appears to be morphologically distinct, with lighter abdominal color than *C. sesamiae* samples of lineages 1 and 3. Genitalia are probably also differentiated, based on the observation that mated pairs between lineage 2 and other lineages had difficulty ending copulation. If lineage 2 were to be identified as a new species, lineages 1 and 3 would form a paraphyletic *C. sesamiae* species. This may be because of a bias linked to the contribution of mtDNA used in our phylogenetic reconstruction. Species-level paraphyly has been found to occur in about 20% of animal species, based on meta-analyses of published mitochondrial gene trees (Ross 2014). The author attributed this problem to a slower rate of mtDNA evolution compared to the rate of species formation. To test the paraphyly of a combined lineages 1 and 3*,* we constrained these 2 lineages to a monophyletic group in a new analysis and compared the results with an unconstrained analysis, using a stepping stones procedure with MrBayes v3.2.1 (Ronquist and Huelsenbeck 2003). The marginal likelihoods was better when lineages 1 and 3 were constrained to monophyly (−7273.37) than when unconstrained (−7322.86). Therefore, lineage 2 could be seen as a new cryptic species of the *flavipes* complex without questioning the integrity of *C. sesamiae* species.

The lineage we studied presents several interesting properties as a potential biological control agent of *S. nonagrioides,* which is a major maize pest in the Mediterranean part of Europe. Its strict specificity for that host (at least in its geographic distribution area) has been established from ecological data, the most reliable way to determine the host range (Brodeur 2012). So risks on nontarget hosts appear unlikely, but cannot be excluded because studies by Barratt et al. (2012) showed that introduced parasitoids could shift on nontarget exotic hosts phylogenetically related to the native hosts. Other advantages are that it is reliably identifiable using molecular markers (Dupas et al. 2006), there are ecological indicators for collecting it from the wild, and reproductive isolation from other populations of the *C*. *flavipes* complex predicts the absence of interference with native parasitoids. In France, *S. nonagrioides* populations have followed maize progression up to the Loire Valley (Rousseau 2009). No biological control agent is yet available against this pest. One species has been considered in Greece, Portugal, and Italy—*Telenomus busseolae* Gahan—but it is an egg parasitoid, a trait often associated with poor host specificity. It may thus threaten nontarget species if released en masse. The lineage *C. sesamiae* Typha is known to be able to develop in European populations of *S. nonagrioides* on maize in laboratory conditions (Kaoula 2009; L. Kaiser, unpublished data). Our study provides a foundation for further developing a program to investigate its potential as a biocontrol agent.

## Data archiving statement

All sequences used in this study have been registered in GenBank. Accession numbers are reported in Table S2. Voucher specimens are preserved at the *icipe* Museum Biosystematic Unit in Nairobi, Kenya.

## Supporting Information

Appendix S1. Methods. **Table S1.** Samples used for the phylogenetic reconstruction of relationships within *C. sesamiae* and in the *flavipes* complex. **Table S2.** PCR reaction conditions. **Figure S1.** Phylogeny of *Cotesia sesamiae* individuals and relatives based on (A) concatenated mtDNA of 3 mitochondrial genes (CO1, 16S, NADH) and nDNA of 1 nuclear non-viral gene (LWRH); (B) concatenated nDNA of two viral genes: EP2 and Histone.
