# Supplementary material for: Ongoing ecological speciation in Cotesia sesamiae, a biological control agent of cereal stem borers
Source: Evol Appl. 2015 Jul 7;8(8):807–20. doi: 10.1111/eva.12260 (PMC4561570; doi:10.1111/eva.12260)
Supplement: Supplementary file 1 [file eva0008-0807-sd1.doc]

**Supplementary material**

Curing *C. sesamiae* of *Wolbachia* infections

Caterpillars parasitized by *C. sesamiae* lab strains were fed on the rearing diet (see Materials and Methods) supplemented with 0.2% (w/v) of the antibiotic rifampicin (Dedeine et al. 2001). The absence of *Wolbachia* was then verified by polymerase chain reaction (PCR) in *C. sesamiae* adults of the first and third generation of rifampicin treatment. DNA was extracted from the whole body of three wasps of each strain reared on treated hosts, and from three reared on untreated hosts as a control, per generation, using a Qiagen DNEasy® extraction kit with an elution volume of 40 µL (Branca et al. 2011b). Four genes were amplified using protocols previously described: *wsp* (Zhou et al. 1998), *gltA*, *groEL* (Casiraghi et al. 2005) and *FtsZ*. For *FtsZ,* primers were redefined from sequences of this gene on GenBank  (*ftsZFDeg*: 5'-AAR-GGT-GCR-GCA GAA-GAR-3' and *FtsZRDeg* : 5'-GCC-YTC-CAT-HGC-YTG-ATY-3'). Gels did not show any amplification of *Wolbachia* DNA in samples of adults that emerged from treated caterpillars, indicating that they had been cured of *Wolbachia* after one generation of rifampicin treatment. Crosses were subsequently performed with insects obtained after one or two generations of antibiotic treatments.

Branca, A., B. P. Le Ru, F. Vavre, J.-F. Silvain and S. Dupas 2011. Intraspecific specialization of the generalist parasitoid *Cotesia sesamiae* revealed by polyDNAvirus polymorphism and associated with different *Wolbachia* infections*.* Molecular Ecology 20: 959-971.

Casiraghi, M., S. R. Bordenstein, L. Baldo et al. 2005. Phylogeny of *Wolbachia pipientis* based on gltA, groEL and ftsZ gene sequences: clustering of arthropod and nematode symbionts in the F supergroup, and evidence for further diversity in the *Wolbachia* tree. Microbiology 151: 4015–4022.

Dedeine, F., F. Vavre, F. Fleury, B. Loppin, M. E. Hochberg and M. Boulétreau 2001. Removing symbiotic *Wolbachia* bacteria specifically inhibits oogenesis in a parasitic wasp. Proceedings of the National Academy of Sciences of the United States of America 98: 6247-6252.

Zhou, W., F. Rousset, S. O’Neill 1998. Phylogeny and PCR-based classification of *Wolbachia* strains using wsp gene sequences. Proceedings of the Royal Society of London: Biological Sciences, 265, 509–515.

| Table S1. Samples used for the phylogenetic reconstruction of relationships within *C. sesamiae* and in the *flavipes* complex. | | | | | | | |
| --- | --- | --- | --- | --- | --- | --- | --- |
| **Sample** | **Host insect** | **Host plant** | **Sp.** | **Locality** | **Lat. E** | **Long. N** | **Sequences** |
| CsK | *Busseola fusca* | *Zea mais* | *Cs* | KE, Kitale | 34.818 | 1.196 | EP2, Hist, CO1, NADH1, LWRH, |
| G4508 | *Pirateolea piscator* | *Pennisetum purpureum* | *Cs* | KE, Kisii_2 | 34.529 | -0.913 | EP2, Hist, CO1, 16S, NADH1, LWRH |
| G4509 | *Schoenobiinae* | *Cyperus imbricatus* | *Cs* | KE, Ruiru | 36.911 | -0.091 | EP2, Hist, 16S, LWRH |
| G4510 | *Schoenobiinae* | *Cyperus imbricatus* | *Cs* | KE, Ruiru | 36.911 | -0.091 | EP2, Hist, CO1, 16S, NADH1, LWRH |
| G4511 | *Schoenobiinae* | *Cyperus imbricatus* | *Cs* | KE, Ruiru | 36.911 | -0.091 | EP2, Hist, CO1, 16S, NADH1, LWRH |
| G4512 | *Sesamia oriaula* | *Pennisetum purpureum* | *Cs* | KE, Kitui | 37.801 | -1.402 | EP2, Hist, CO1, 16S, LWRH |
| G4540 | *Busseola fusca* | *Sorghum arundinaceum* | *Cs* | KE, Mombasa | 37.490 | -2.091 | EP2, Hist, CO1, NADH1, LWRH |
| G4559 | *Chilo partellus* | *Sorghum arundinaceum* | *Cs* | KE, Rhamisi | 39.407 | -4.523 | EP2, Hist, CO1, 16S, NADH1, LWRH |
| G4560 | *Chilo sp.* | *Panicum maximum* | *Cs* | TZ, Zb, Potoa | 39.283 | -5.849 | EP2, Hist, 16S, NADH1, LWRH |
| G4561 | *Chilo orichalcociliellus* | *Panicum maximum* | *Cs* | TZ, Zb, Kizimbani | 39.257 | -6.086 | EP2, Hist, CO1, 16S, NADH1, LWRH |
| G4572 | *Busseola phaia* | *Pennisetum purpureum* | *Cs* | TZ, Mafinga | 35.307 | -8.302 | EP2, Hist, CO1, 16S, NADH1, LWRH |
| G4579 | *Chilo partellus* | *Pennisetum purpureum* | *Cs* | TZ, Zb, Kizimbani | 39.257 | -6.086 | EP2, Hist, NADH1, LWRH |
| G4583 | *Pirateolea piscator* | *Cyperus dives* | *Cs* | TZ, Kimamba | 36.088 | -7.661 | EP2, Hist, 16S, NADH1, LWRH |
| G4594 | *Busseola fusca* | *Arundo donax* | *Cs* | ER, Adi Boskal | 38.648 | 15.695 | EP2, Hist, CO1, 16S, NADH1, LWRH |
| G4602 | *Sesamia nonagrioides* | *Pennisetum purpureum* | *Cs* | KE, Kisii_2 | 34.529 | -0.913 | EP2, Hist, CO1, 16S, NADH1, LWRH |
| G4608 | *Sesamia nonagrioides* | *Typha domingensis* | *Cs* | ET, Omolante | 37.667 | 6.158 | EP2, Hist, CO1, NADH1, LWRH |
| G4609 | *Sesamia nonagrioides* | *Typha domingensis* | *Cs* | ET, Omolante | 37.667 | 6.158 | EP2, Hist, CO1, 16S, NADH1, LWRH |
| G4610 | *Sesamia nonagrioides* | *Typha domingensis* | *Cs* | ET, Omolante | 37.667 | 6.158 | EP2, Hist, CO1, 16S, NADH1, LWRH |
| G4612 | *Sesamia nonagrioides* | *Typha domingensis* | *Cs* | ET, Omolante | 37.667 | 6.158 | EP2, Hist, CO1, 16S, NADH1, LWRH |
| G4613 | *Sesamia nonagrioides* | *Typha domingensis* | *Cs* | ET, Awasa | 38.468 | 7.054 | EP2, Hist, CO1, 16S, NADH1, LWRH |
| G4614 | *Sesamia nonagrioides* | *Typha domingensis* | *Cs* | ET, Awasa | 38.468 | 7.054 | EP2, Hist, CO1, 16S, NADH1, LWRH |
| G4615 | *Sesamia nonagrioides* | *Typha domingensis* | *Cs* | ET, Awasa | 38.468 | 7.054 | EP2, Hist, CO1, 16S, NADH1, LWRH |
| G4616 | *Sesamia nonagrioides* | *Typha domingensis* | *Cs* | ET, Awasa | 38.468 | 7.054 | EP2, Hist, CO1, 16S, NADH1, LWRH |
| G4617 | *Sesamia nonagrioides* | *Typha domingensis* | *Cs* | ET, Chamoleto | 37.534 | 5.926 | EP2, Hist, 16S, NADH1, LWRH |
| G4618 | *Sesamia nonagrioides* | *Typha domingensis* | *Cs* | ET, Chamoleto | 37.534 | 5.926 | EP2, Hist, CO1, 16S, NADH1, LWRH |
| G4619 | *Sesamia nonagrioides* | *Typha domingensis* | *Cs* | ET, Chamoleto | 37.534 | 5.926 | EP2, Hist, CO1, 16S, NADH1, LWRH |
| G4636 | *Busseola s.l. 1 nov. sp. 1* | *Setaria megaphylla* | *Cs* | KE, Kakamega | 34.894 | 0.375 | EP2, Hist, CO1, 16S, NADH1, LWRH |
| G4652 | *Sesamia firmata* | *Pennisetum purpureum* | *Cs* | KE, Tana | 37.265 | -0.789 | EP2, Hist, CO1, 16S, LWRH |
| G4655 | *Sesamia nonagrioides* | *Typha domingensis* | *Cs* | KE, Mbita Lwanda | 34.297 | -0.483 | EP2, Hist, CO1, 16S, LWRH |
| G4656 | *Sesamia nonagrioides* | *Typha domingensis* | *Cs* | KE, Mbita Lwanda | 34.297 | -0.483 | EP2, Hist, CO1, 16S, NADH1, LWRH |
| G4659 | *Sesamia penniseti* | *Pennisetum purpureum* | *Cs* | KE, Kisii 2 | 34.529 | -0.913 | EP2, Hist, 16S, NADH1, LWRH |
| G4664 | *Sesamia nonagrioides* | *Typha domingensis* | *Cs* | KE, Mbita Lwanda | 34.297 | -0.483 | EP2, Hist, CO1, 16S, NADH1, LWRH |
| G4665 | *Sesamia nonagrioides* | *Typha domingensis* | *Cs* | KE, Mbita Lwanda | 34.297 | -0.483 | EP2, Hist, CO1, 16S, NADH1, LWRH |
| G4666 | *Sesamia nonagrioides* | *Typha domingensis* | *Cs* | KE, Mbita Lwanda | 34.297 | -0.483 | EP2, Hist, CO1, 16S, NADH1, LWRH |
| G4667 | *Sesamia nonagrioides* | *Typha domingensis* | *Cs* | KE, Mbita Lwanda | 34.297 | -0.483 | EP2, Hist, CO1, 16S, NADH1, LWRH |
| G4668 | *Sesamia nonagrioides* | *Typha domingensis* | *Cs* | KE, Mbita Lwanda | 34.297 | -0.483 | EP2, Hist, 16S, NADH1, LWRH |
| G4669 | *Sesamia nonagrioides* | *Typha domingensis* | *Cs* | KE, Mbita Lwanda | 34.297 | -0.483 | EP2, Hist, CO1, 16S, NADH1, LWRH |
| G4670 | *Sesamia nonagrioides* | *Typha domingensis* | *Cs* | KE, Mbita Lwanda | 34.297 | -0.483 | EP2, Hist, 16S, NADH1, LWRH |
| G4672 | *Sesamia nonagrioides* | *Typha domingensis* | *Cs* | KE, Mbita Lwanda | 34.297 | -0.483 | EP2, Hist, 16S, NADH1, LWRH |
| G4675 | *Sesamia nonagrioides* | *Typha domingensis* | *Cs* | KE, Mbita Lwanda | 34.297 | -0.483 | EP2, Hist, CO1, 16S, NADH1, LWRH |
| G4676 | *Sesamia nonagrioides* | *Typha domingensis* | *Cs* | KE, Mbita Lwanda | 34.297 | -0.483 | EP2, Hist, CO1, 16S, NADH1, LWRH |
| G4677 | *Sesamia nonagrioides* | *Typha domingensis* | *Cs* | KE, Mbita Lwanda | 34.297 | -0.483 | EP2, Hist, CO1, 16S, NADH1, LWRH |
| G4678 | *Sesamia nonagrioides* | *Typha domingensis* | *Cs* | KE, Mbita Lwanda | 34.297 | -0.483 | EP2, Hist, CO1, 16S, NADH1, LWRH |
| G4689 | *Sesamia calamistis* | *Sorghum bicolor* | *Cs* | KE, Kitale | 34.818 | 1.196 | EP2, Hist, CO1, 16S, NADH1, LWRH |
| G4691 | *Sesamia calamistis* | *Sorghum bicolor* | *Cs* | KE, Kitale | 34.818 | 1.196 | EP2, Hist, CO1, 16S, NADH1, LWRH |
| G4692 | *Sesamia calamistis* | *Sorghum bicolor* | *Cs* | KE, Kitale | 34.818 | 1.196 | EP2, Hist, 16S, NADH1, LWRH |
| G4701 | *Chilo sp.* | *Echinochloa pyramidalis* | *Cs* | UG, Namasoga | 33.423 | 0.570 | EP2, Hist, CO1, 16S, NADH1, LWRH |
| G4703 | *Pirateolea piscator* | *Cyperus dives* | *Cs* | KE, Githurai | 37.2 | -0.717 | EP2, Hist, CO1, 16S, NADH1, LWRH |
| G4708 | *Crambidae* | *Echinochloa pyramidalis* | *Cs* | KE, Homa Bay | 34.535 | -0.673 | EP2, Hist, CO1, 16S, NADH1, LWRH |
| G4907 | *Busseola s.l. 2 nov. sp. 1* | *Pennisetum trachyphyllum* | *Cs* | KE, Moutain lodge | 37.150 | -0.317 | EP2, Hist, CO1, 16S, NADH1, LWRH |
| G4909 | *Sesamia nonagrioides* | *Typha domingensis* | *Cs* | ET, Omolante | 37.667 | 6.158 | EP2, Hist, CO1, 16S, NADH1, LWRH |
| G4915 | *Busseola s.l. 2 nov. sp. 1* | *Cymbopogon nardus* | *Cs* | KE, Chepsir | 35.431 | -0.282 | EP2, Hist, CO1, 16S, NADH1, LWRH |
| G4916 | *Sesamia calamistis* | *Cyperus dives* | *Cs* | KE, Suam | 34.877 | 1.067 | EP2, Hist, 16S, NADH1, LWRH |
| G5699 | *Sesamia firmata* | *Pennisetum purpureum* | *Cs* | KE, Tana | 37.265 | -0.789 | EP2, Hist, CO1, 16S, NADH1, LWRH |
| G5726 | *Sesamia nonagrioides* | *Typha domingensis* | *Cs* | KE, Mbita Lwanda | 34.297 | -0.483 | EP2, Hist, CO1, 16S, NADH1, LWRH |
| G5773 | *Sesamia nonagrioides* | *Typha domingensis* | *Cs* | KE, Masimba | 37.582 | -2.152 | EP2, Hist, CO1, NADH1, LWRH |
| G5775 | *Busseola phaia* | *Pennisetum purpureum* | *Cs* | TZ, Ndanda | 39.017 | -10.502 | EP2, Hist, CO1, NADH1, LWRH |
| G5776 | *Sesamia nonagrioides* | *Cyperus dives* | *Cs* | TZ, Ruvu | 38.708 | -6.7009 | EP2, Hist, CO1, 16S, NADH1, LWRH |
| G5777 | *Busseola fusca* | *Cymbopogon nardus* | *Cs* | TZ, Tokera | 33.556 | -9.096 | EP2, Hist, 16S, NADH1, LWRH |
| G5778 | *Busseola s.l. 2 nov. sp. 2* | *Pennisetum unisetum* | *Cs* | TZ, Waro Kolobo | 36.833 | -7.614 | EP2, Hist, CO1, 16S, NADH1, LWRH |
| G5780 | *Pirateola sp* | *Cyperus digitatus* | *Cs* | TZ, Makala | 37.243 | -10.912 | EP2, Hist, 16S, NADH1, LWRH |
| G7313 | *Pirateolea piscator* | *Pennisetum macrourum* | *Cs* | TZ, Nzi | 35.690 | -7.716 | EP2, Hist, 16S, NADH1, LWRH |
| G7338 | *Manga nubifera* | *Panicum maximum* | *Cs* | TZ, Mwaya | 36.817 | -8.917 | EP2, Hist, CO1, 16S, NADH1, LWRH |
| G9010 | *Sesamia nonagrioides* | *Cyperus dives* | *Cs* | KE, Kisumu Rabuor | 34.775 | -0.110 | EP2, Hist, CO1, 16S, NADH1, LWRH |
| G9093 | *Sesamia nonagrioides* | *Cyperus dives* | *Cs* | KE, Kabuto | 34.170 | -0.924 | EP2, Hist, CO1, 16S, NADH1, LWRH |
| G9098 | *Sesamia nonagrioides* | *Cyperus dives* | *Cs* | KE, Kabuto | 34.170 | -0.924 | EP2, Hist, CO1, 16S, NADH1, LWRH |
| G9103 | *Sesamia nonagrioides* | *Cyperus dives* | *Cs* | KE, Kobodo | 34.412 | -0.679 | EP2, Hist, CO1, 16S, NADH1, LWRH |
| G9111 | *Sesamia nonagrioides* | *Cyperus dives* | *Cs* | KE, Kobodo | 34.412 | -0.679 | EP2, Hist, CO1, 16S, NADH1, LWRH |
| Kitale2 | *Busseola fusca* | *Sorghum arundinaceum* | *Cs* | KE, Kitale | 34.818 | 1.196 | Hist, 16S, NADH1, LWRH |
| Mbita | *Sesamia nonagrioides* | *Typha domingensis* | *Cs* | KE, Mbita Lwanda | 34.297 | -0.483 | EP2, Hist, CO1, 16S, NADH1, LWRH |
| MbL | *Sesamia nonagrioides* | *unknown* | *Cs* | KE, Mbita Lwanda | 34.297 | -0.483 | EP2, Hist, CO1, NADH1, LWRH |
| Mhk | *Sesamia calamistis* | *Zea mais* | *Cs* | KE, Muhaka | 39.5 | -4.33 | EP2, Hist, CO1, NADH1, LWRH |
| Mkd | *Sesamia nonagrioides* | *Typha domingensis* | *Cs* | KE, Makindu | 37.825 | -2.278 | EP2, Hist, CO1, NADH1, LWRH |
| P0433 | *unknown* | *unknown* | *Cf* | South Pakistan | unknown | unknown | EP2, Hist, CO1, 16S, NADH1, LWRH |
| P0434 | *unknown* | *unknown* | *Cf* | North Pakistan | unknown | unknown | EP2, Hist, CO1, 16S, NADH1, LWRH |
| P0435 | *unknown* | *unknown* | *Cf* | India | unknown | unknown | EP2, Hist, CO1, 16S, NADH1, LWRH |
| P2541 | *unknown* | *unknown* | *Cf* | KE, Mombasa | 37.490 | -2.091 | EP2, Hist, CO1, 16S, NADH1, LWRH |
| P4697 | *Busseola phaia* | *Pennisetum purpureum* | *Cs* | MZ, Rio_chiteo | 32.82 | -18.969 | EP2, Hist, 16S, NADH1, LWRH |
| P4706 | *Busseola fusca* | *Zea mais* | *Cf* | KE, Makindu | 37.825 | -2.278 | EP2, Hist, CO1, 16S, NADH1, LWRH |
| P6679 | *Chilo suppressalis* | *Oriza sativa* | *Cc* | JA, Shiga | 135.871 | 35.006 | EP2, Hist, CO1, 16S, NADH1, LWRH |
| P6680 | *Chilo suppressalis* | *Oriza sativa* | *Cc* | JA, Shiga | 135.870 | 35.005 | EP2, Hist, CO1, 16S, NADH1, LWRH |
| P6681 | *Chilo suppressalis* | *Oriza sativa* | *Cc* | JA, Shiga | 135.869 | 35.004 | EP2, Hist, CO1, NADH1, LWRH |

Sample reference codes correspond to the data bank of the Laboratoire Evolution, Génome et Spéciation. *Cs: Cotesia sesamiae; Cf: C. flavipes*. KE: Kenya, TZ: Tanzania, ER: Eritrea, ET: Ethiopia, UG: Uganda, MZ: Mozambique, JA: Japan. EP2 (Early Expressed) and Hist (Histone4): nuclear genes of the polyDNA virus; LWRH (Long-Wavelength Rhodopsin ): non-viral nuclear gene; CO1, NADH1 and 16S: mtDNA.

Table S2: PCR reaction conditions.

| Gene | Accession  numbers | Primer name | Sequence | Primer (µM) | MgCl2 (mM) | dNTP (mM) | Tm (°C) | Cycles | References |
| --- | --- | --- | --- | --- | --- | --- | --- | --- | --- |
| Histone H4 | KJ882627 to  KJ882701,  KP100090 to  KP100096 | HistCs-F  HistCs-R | ATGTCTGATTGTCCTAAAGAT  TCAACCTCCATAACCATAGAT | 0.4 | 4 | 0.4 | 60 | 5'95°C ;  40×(1'95°C ;1'30"60°C ;1' 72°C) ;  5' 72°C | This study |
| EP2 | KJ882552 to  KJ882626,  KP100083 to  KP100089 | EP2Cs-F2  EP2Cs-R2 | CTAAGCAGAAGAACTTCTTC  TCAGTTGCGTTTAACTCG | 0.4 | 4 | 0.4 | 60 | 5'95°C ;  40×(1'95°C ;1'30"60°C ;1' 72°C) ;  5' 72°C | This study |
| NADH1 | KJ882777 to  KJ882847,  KP100104 to  KP100110 | ND1F  ND1R | ACTAATTCAGATTCTCCTTCT  CAACCTTTTAGTGATGC | 0.4 | 4 | 0.4 | 50 | 3'95°C ;  40×(50"95°C ;1'30"50°C ;1' 72°C) ;  7' 72°C | Smith et al. (1999) |
| COI | KJ882493 to  KJ882551,  KP100076 to  KP100082 | LCO  HCO | GGTCAACAAATCATAAAGATATTGG  TAAACTTCAGGGTGACCAAAAAATCA | 0.4 | 3 | 0.4 | 50 | 5'94°C ;  40×(1'94°C ;1'30"50°C ;1' 72°C) ;  5' 72°C | Folmer et al. (1994) |
| 16S | KJ882429 to  KJ882492,  KP100070 to  KP100075 | LRJ-12887  LRN-13398 | TTACGCTGTTATCCCTAA  CGCCTGTTTATCAAAAACAT | 0.4 | 4 | 0.4 | 50 | 5'95°C ;  40×(1'95°C ;1'30"50°C ;1' 72°C) ;  5' 72°C | Simon et al. (1994) |
| LWRH | KJ882702 to  KJ882776,  KP100097 to  KP100103 | LWRH_Cot  LWRH_Cot | GTGCTGGTTCCCTGTTCGGATG  GCAAGTTTGCAYTCAGCGCTTTG | 0.4 | 3 | 0.4 | 58 | 5'95°C ;  40×(1'95°C ; 1'30"58°C ;1' 72°C) ;  5' 72°C | This study |

Dupas, S., C. W. Gitau, A. Branca, B. P. Le Ru and J.-F. Silvain 2008. Evolution of a polydnavirus gene in relation to parasitoid-host species immune resistance. Journal of Heredity 99: 491–499.

Folmer, O., M. Black, W. Hoeh, R. Lutz and R. Vrijenhoek 1994. DNA primers for amplification of mitochondrial cytochrome. Molecular Marine Biology *and* Biotechnology 3: 294–299.

Smith, P.T., S. Kambhampati, W. Völkl and M. Mackauer 1999. A phylogeny of aphid parasitoids (Hymenoptera: Braconidae: Aphidiinae) inferred from mitochondrial NADH 1 dehydrogenase gene sequence. Molecular Phylogenetic Evolution 11: 236–45.

Simon, C., F. Frati, A. Beckenbach, B. Crespi, H. Liu and P. Flook 1994. Evolution, weighting, and phylogenetic utility of mitochondrial gene sequences and a compilation of conserved polymerase chain-reaction primers. Annals of the Entomological Society of America 87: 651e701.


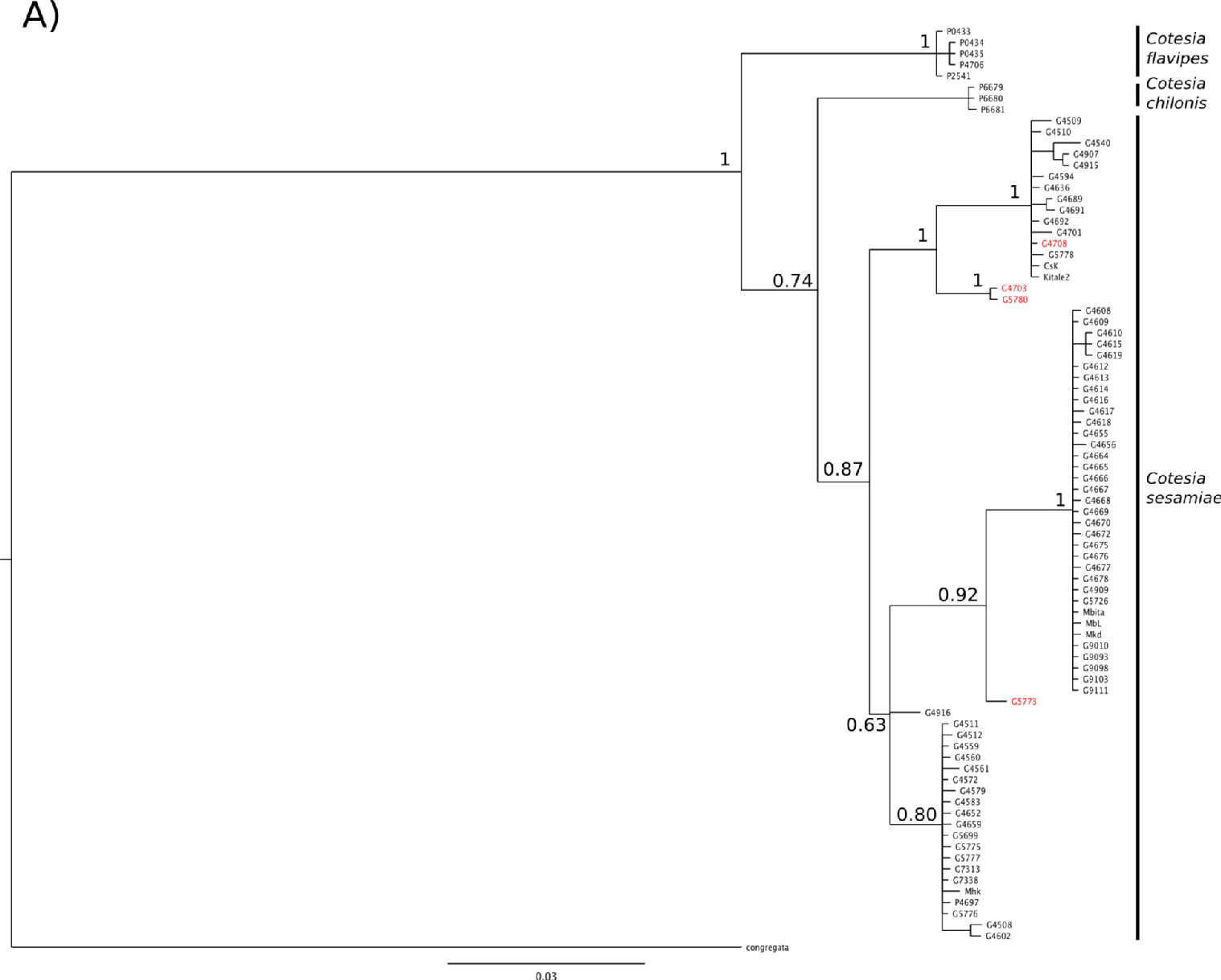


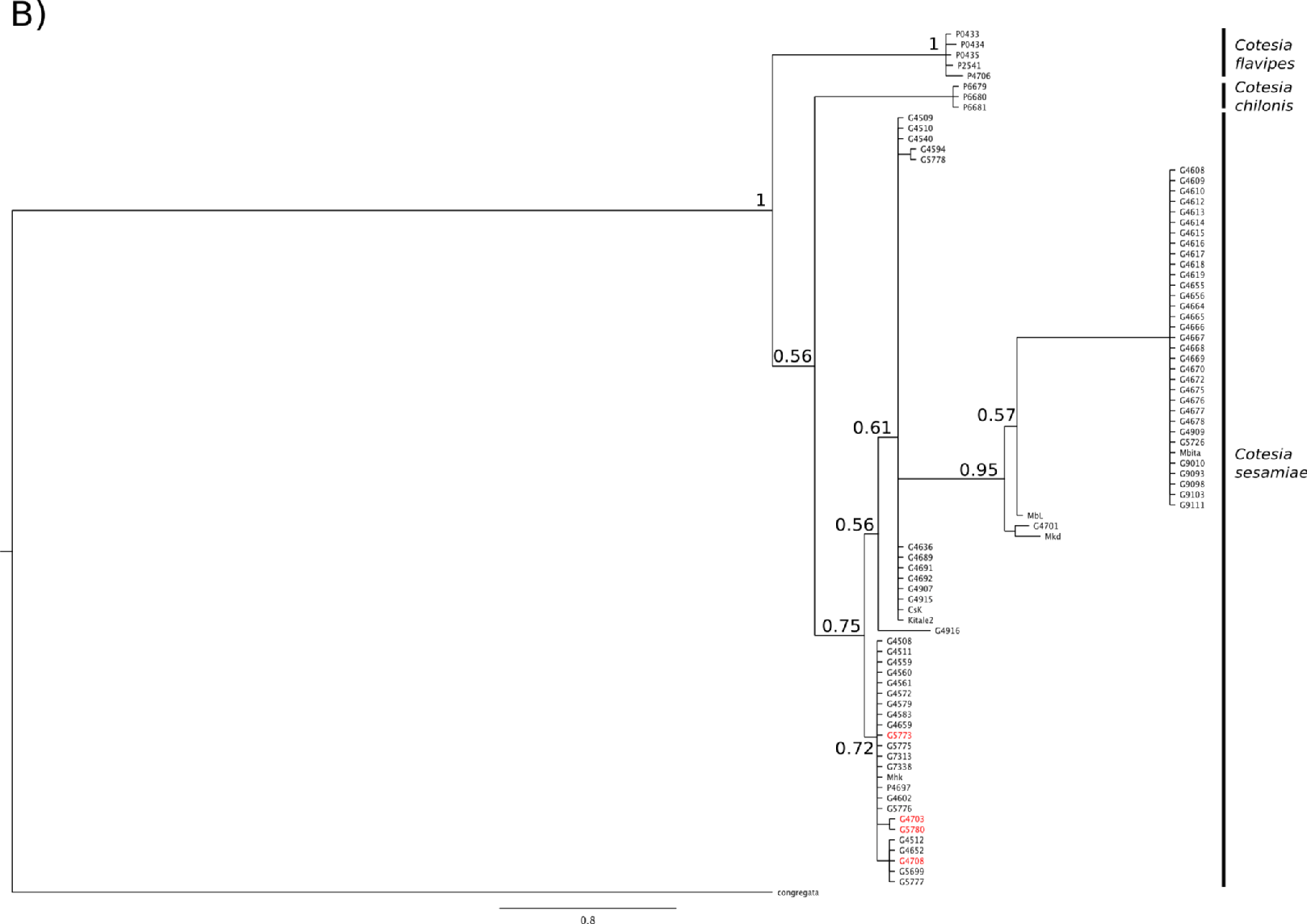


Figure S1. Phylogeny of *Cotesia sesamiae* individuals and relatives based on (A) concatenated mtDNA of 3 mitochondrial genes (CO1, 16S, NADH) and nDNA of 1 nuclear non-viral gene (LWRH); (B) concatenated nDNA of two viral genes: EP2 and Histone. See Materials and Methods for substitution model selection with PartitionFinder and phylogenetic tree inference in Mr Bayes. Posterior probabilities are given at nodes. All samples have a reference code corresponding to the data bank of the Laboratoire Evolution, Génome, Comportement et Ecologie. Samples in red are those with incongruent phylogenetic positioning compared to the topology produced by the 6-genes based phylogenetic analysis (Fig. 1)
